# Comparison of blood RNA isolation methods from samples stabilized in Tempus tubes and stored at a large human biobank

**DOI:** 10.1186/s13104-016-2224-y

**Published:** 2016-09-01

**Authors:** Jeanette Aarem, Gunnar Brunborg, Kaja K. Aas, Kari Harbak, Miia M. Taipale, Per Magnus, Gun Peggy Knudsen, Nur Duale

**Affiliations:** 1 Norwegian Institute of Public Health, P.O Box 4404, 0403 Nydalen, Oslo, Norway; 2 Institute for Energy Technology, Kjeller, Norway

**Keywords:** Tempus tubes, Cord blood, MiRNA, Noncoding RNA, Gene expression, RNA isolation, Epigenetics, The Norwegian Mother and Child Cohort Study, MoBa, Biobank

## Abstract

**Background:**

More than 50,000 adult and cord blood samples were collected in Tempus tubes and stored at the Norwegian Institute of Public Health Biobank for future use. In this study, we systematically evaluated and compared five blood-RNA isolation protocols: three blood-RNA isolation protocols optimized for simultaneous isolation of all blood-RNA species (MagMAX RNA Isolation Kit, both manual and semi-automated protocols; and Norgen Preserved Blood RNA kit I); and two protocols optimized for large RNAs only (Tempus Spin RNA, and Tempus 6-port isolation kit). We estimated the following parameters: RNA quality, RNA yield, processing time, cost per sample, and RNA transcript stability of six selected mRNAs and 13 miRNAs using real-time qPCR.

**Findings:**

Whole blood samples from adults (n = 59 tubes) and umbilical cord blood (n = 18 tubes) samples collected in Tempus tubes were analyzed. High-quality blood-RNAs with average RIN-values above seven were extracted using all five RNA isolation protocols. The transcript levels of the six selected genes showed minimal variation between the five protocols. Unexplained differences within the transcript levels of the 13 miRNA were observed; however, the 13 miRNAs had similar expression direction and they were within the same order of magnitude. Some differences in the RNA processing time and cost were noted.

**Conclusions:**

Sufficient amounts of high-quality RNA were obtained using all five protocols, and the Tempus blood RNA system therefore seems not to be dependent on one specific RNA isolation method.

**Electronic supplementary material:**

The online version of this article (doi:10.1186/s13104-016-2224-y) contains supplementary material, which is available to authorized users.

## Findings

### Background

Blood-based biobanks such as the NIPH Biobank at the Norwegian Institute of Public Health (NIPH), and multi-center studies, increasingly incorporate studies identifying candidate mRNA and microRNA (miRNA) expression profile based biomarkers for a wide range of disorders. Identification of biomarkers in an easily accessible and minimally-invasive biological sample such as blood would be a valuable complementing tool for diagnostics and prognostics of different types of diseases. The strong stability of miRNAs in circulating blood and their role as key regulators of almost every biological process [1] suggests that they could serve as non-invasive biomarkers for a wide range of diseases [2–5]. Furthermore, miRNAs have been shown to be targeted by epigenetic modification, and in turn, miRNAs can target regulators of epigenetic pathways [6–9]. MiRNAs’ role in neurodevelopmental diseases, both as diagnostic biomarkers as well as explaining basic disease etiology has come into focus; aberrant miRNA function has been linked to the etiology of several neurological disorders [10–15]. Recently a set of five miRNAs in blood serum of children with autism spectrum disorder (ASD) was suggested as potential candidates for circulating miRNA-based prediction of ASD [16].

The Norwegian Mother and Child Cohort Study (MoBa) is a prospective population-based pregnancy cohort study conducted by the NIPH. Participants were recruited from all over Norway from 1999 to 2008, and the cohort now includes more than 114,500 children, 95,200 mothers and 75,200 fathers [17]. Biological material in the form of whole blood and plasma has been collected from the mother, the father and the child (umbilical cord blood) and stored in the Biobank, together with extracted DNA, for future use [18]. In 2005, as part of the Autism Birth Cohort (ABC) study with support from the National Institute of Neurological Disorders and Stroke (NINDS), we evaluated two commercially available RNA stabilizing technologies for the collection of blood in MoBa: PAXgene Blood RNA system (PreAnalytix, QIAGEN/BD) and Tempus Blood RNA system (Life Technologies) [19]. Since 2005, more than 50,000 adult and cord blood samples were collected in the Tempus tubes and stored in the NIPH Biobank for future RNA isolation and downstream analyses.

Recently, we have systematically evaluated a blood-RNA isolation protocol for blood samples collected in the Tempus tubes using Tempus 6-port RNA isolation kit (Life Technologies) on a 6100 Nucleic Acid Prep Station [19, 20]. This protocol is now a well-established blood-RNA isolation protocol at the NIPH Biobank. However, the Tempus 6-port RNA isolation kit has some limitations; in particular, it does not retain well small RNAs (<200 nucleotides), i.e., most of the small noncoding RNAs (ncRNAs) will be lost during the RNA isolation. Recently, the Tempus tube supplier (Life Technologies, Norway), and Norgen Biotek Corp (Norgen Biotek Corp, Canada) have introduced new blood-RNA isolation kits optimized for simultaneous isolation of all RNA species from blood samples stabilized in Tempus tubes. To make the NIPH Biobank more flexible and not to be dependent on one specific RNA isolation method, it is very important to establish several comparable blood-RNA isolation protocols. Consequently, the NIPH Biobank has established alternative blood-RNA isolation protocols capable of isolating all RNA species simultaneously from the blood samples collected in the Tempus tubes.

In this study, we report on the evaluation of five blood-RNA isolation protocols: MagMAX semi-automated, MagMAX manual, Tempus spin, Tempus 6-port, and Norgen (Table 1). These protocols are optimized for blood samples collected in Tempus tubes. The protocols were evaluated and compared with regard to their suitability of isolating high-quality RNA (including small ncRNAs) from adult and cord blood samples collected in Tempus tubes and stored at −80 °C at the NIPH Biobank. The performance of the methods, i.e. the sample processing time and cost, and the possibility of (semi)-automation of the sample processing part, have to be considered when introducing a new RNA isolation method. Here, we report a systematic evaluation and comparison of five blood-RNA isolation protocols, with regards to RNA quality (i.e., purity and integrity), yield, RNA processing time, cost per sample, and RNA transcript stability of six genes (*CDKN1A*, *FOS*, *IL1B*, *IL8*, *MYC, TP53*) and 13 miRNAs (*hsa*-*let*-*7a, hsa*-*miR*-*16, hsa*-*miR*-*20a, hsa*-*miR*-*21, hsa*-*miR*-*26a, hsa*-*miR*-*34a, hsa*-*miR*-*451, hsa*-*miR*-*93, hsa*-*miR*-*103, hsa*-*miR*-*126, hsa*-*miR*-*191, hsa*-*miR*-*192, hsa*-*miR*-*423*-*3p*) selected based on our previous studies [19, 20] and literature search [21, 22]. Furthermore, the new protocols were compared against the Tempus 6-port protocol which is already a well-established RNA isolation protocol at the Biobank [19, 20].

**Table 1:** Overview of the five RNA isolation protocols

RNA isolation protocol	RNA isolation kit name	RNA processing method	Technologies for tot RNA extraction	Simultenous isolation of all RNA species^c^
MagMAX semi-automated^a^	MagMAX™ for Stabilized Blood Tubes RNA Isolation Kit, compatible with Tempus™ Blood RNA Tubes	Semi-automated	Magnetic beads based RNA purification system	Yes
MagMAX manual^a^	MagMAX™ for Stabilized Blood Tubes RNA Isolation Kit, compatible with Tempus™ Blood RNA Tubes	Manual	Magnetic beads based RNA purification system	Yes
Norgen^b^	Preserved Blood RNA Purification Kit I (for use with Tempus Blood RNA Tubes)	Manual	Column-based RNA purification systems:resin as the separation matrix	Yes
Tempus Spin^a^	Tempus™ Spin RNA Isolation Kit	Manual	Column-based RNA purification systems:silica membrane	No
Tempus 6-port^a^	Tempus™ 6-Port RNA Isolation Kit	Semi-automated	Column-based RNA purification systems:silica membrane	No

^a^The kit supplier is Life Technologies, Norway

^b^The kit supplier is Norgen Biotek Corp, Canada

^c^All RNA species can be simultaneously isolated according to the manufacturer’s protocol

### Results and discussion

In MoBa, the combination of biological specimens and questionnaire data on lifestyle and exposures provide unique possibilities to study the effects of many factors of relevance for pregnancy outcomes and health. In order to get more insight into the biological mechanisms triggered by gene-environment interactions and disease, the NIPH Biobank is now incorporating blood-based mRNA and miRNA expression profiling studies. However, reliable quantification of mRNA and miRNA levels requires high-quality RNA, and compromised RNA integrity has been shown to influence the quantification of mRNA and miRNA levels [23–25]. Recently, we reported that intact and high-quality RNA suitable for mRNA profiling analyses was obtained from blood samples collected in the Tempus tubes and stored at −80 °C, of satisfactory stability during storage over a period of up to 6 years [20]. However, the well-established RNA isolation protocol (Tempus 6-port RNA isolation kit on a 6100 Nucleic Acid Prep Station) at NIPH Biobank, is not optimized for isolation of small RNA molecules (<200 nucleotides). Therefore, a comparison and evaluation of several blood-RNA isolation protocols which may replace the well-established Tempus 6-port protocol at the NIPH Biobank was conducted.

To evaluate the RNA quality and transcript stability of the blood-RNAs isolated using five blood-RNA isolation protocols (Table 1), blood samples collected in the Tempus tubes and stored at −80 °C, were thawed according to the manufacturers’ recommendations. Total RNA was isolated from adult blood (n = 59 Tempus tubes from four donors) and cord blood (n = 18 Tempus tubes from six donors) samples using the five RNA isolation protocols. For each blood-RNA isolation protocol, 11–12 Tempus tubes containing adult blood and 3–6 Tempus tubes containing cord blood were analyzed. The RNA yield and purity were measured by spectroscopic quantification using NanoDrop ND-8000 Spectrophotometer. The RNA integrity, expressed as RIN values, was assessed using an Agilent 2100 Bioanalyzer, and the transcript stability for six target mRNAs and 13 target miRNAs were analyzed by real-time qPCR assay.

**Fig. 1:**
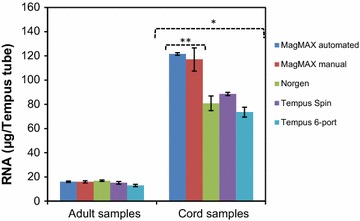
Comparison of RNA yields for the adult and cord blood samples collected in Tempus tubes. The RNA yield from adult blood samples (n = 12 Tempus tubes per protocol, n = 11 Tempus tubes for the Tempus 6-port protocol) and the RNA yield from cord blood samples (n = 3 Tempus tubes per protocol, except the ABI system, where n = 6 Tempus tubes). *The RNA yields from cord blood samples were significantly higher than for adult blood samples, and **the RNA yields obtained from cord blood samples using the two MagMAX protocols were significantly higher than the RNA yields obtained using the other three protocols (p < 0.05). *Each bar* represents the average RNA yield and the *error bars* indicate ± SE

The average total RNA yield for the adult and cord blood samples of the five RNA isolation protocols were 15.4 ± 4.2 and 90.8 ± 23.6 μg per Tempus tube, respectively (Fig. 1). No significant differences in the average RNA yield between the five protocols were observed from adult blood samples (Fig. 1). Similar to our previous reports [19, 20], the RNA yields obtained from the cord blood samples were significantly higher than the RNA yield obtained from the adult blood samples (Fig. 1). For cord blood samples, statistically significantly higher RNA yields were obtained from blood samples isolated using the two MagMAX (semi-automated and manual) protocols (p < 0.05), compared to the other three protocols (Fig. 1). The reason for this is unknown but may be due to the RNA isolation technology using magnetic beads for capturing the RNA. However, no such protocol-related difference was observed for adult blood. The amounts of RNA obtained from both adult and cord blood samples from all five protocols were within the range of RNA yields reported previously [20, 26], and the obtained yields of RNA were sufficient for downstream analysis.

The integrity and purity of RNA can be used to evaluate the performance of the RNA isolation protocols. RNA with an OD 260/280 ratio >1.9 are generally accepted as pure RNA suitable for gene expression analyses [27], and OD 260/230 ratio <1.8 generally indicates the presence of contaminants. The OD 260/280 and OD 260/230 ratios of the isolated total RNA from adult and cord blood samples are shown in Table 2. The average OD 260/280 ratios for adult and for cord blood samples were 2.12 ± 0.01 and 2.10 ± 0.02, respectively, indicating RNA of good quality (Table 2). There are no significant differences between the RNA isolation protocols, and the OD 260/280 ratios for the samples were within an acceptable range of high-quality RNAs. However, the average OD 260/230 ratios from adult blood samples isolated with the MagMAX manual and the Norgen protocols were significantly lower (p < 0.05) than the OD 260/230 ratios from samples isolated with the other protocols (Table 2a). The reason for the observed differences is unclear. High salt content in the elution buffer may have more influence on the OD 260/230 ratios when the RNA amount is low. This difference was not observed for the cord blood samples, for which the average OD 260/230 ratios were above 2.0 (Table 2b), indicating good quality RNA.

**Table 2:** Comparison of RNA QC using five RNA isolation protocols

RNA isolation protocol	RIN value	OD 260/280 ratio	OD 260/230 ratio	Number of Tempus tubes
(a) Adult blood				
MagMAX semi-automated	7.85 ± 0.13	2.09 ± 0.01	1.97 ± 0.05	12
MagMAX manual	7.20 ± 0.11	2.12 ± 0.01	1.19 ± 0.12*	12
Norgen	7.36 ± 0.12	2.12 ± 0.02	1.76 ± 0.07*	12
Tempus spin	8.97 ± 0.05	2.09 ± 0.01	2.08 ± 0.01	12
Tempus 6-port	8.38 ± 0.08	2.20 ± 0.06	2.02 ± 0.05	11
Average	7.93 ± 0.10	2.12 ± 0.01	1.80 ± 0.05	59
(b) Cord blood				
MagMAX semi-automated	7.47 ± 0.22	2.09 ± 0.03	2.01 ± 0.09	3
MagMAX manual	7.73 ± 0.47	2.12 ± 0.01	2.14 ± 0.02	3
Norgen	8.23 ± 0.33	2.11 ± 0.01	2.07 ± 0.04	3
Tempus spin	7.13 ± 0.13	2.10 ± 0.01	2.11 ± 0.01	3
Tempus 6-port	8.37 ± 0.38	2.10 ± 0.01	2.10 ± 0.02	6
Average	7.88 ± 0.19	2.10 ± 0.02	2.09 ± 0.05	18

* RNA extracted with MagMAX manual and Norgen protocols have significantly lower OD 260/230 ratio compared to RNA from the other protocols, p < 0.05

The RNA integrity, expressed as RIN values, of the RNA samples from each RNA isolation protocol, was calculated using the Bioanalyzer (Table 2). The average RIN value for each protocol for both adult blood and cord blood samples was around seven or higher, which reflects high-quality RNA; RIN values above seven are considered acceptable for most gene expression profiling methods [23, 25, 27]. However, there were some, but not significant, differences in the average RIN values between the protocols. For adult blood samples, the RNA samples isolated using Tempus Spin had the highest average RIN values (8.97 ± 0.05) and samples isolated using MagMAX manual had the lowest average RIN values (7.20 ± 0.11) (Table 2a). For cord blood samples, the RNA samples isolated using Tempus 6-port had the highest average RIN values (8.37 ± 0.38), while samples isolated using Tempus Spin had the lowest average RIN values (7.13 ± 0.13) (Table 2b). The observed RIN values were comparable between the protocols and they were within the range of the RIN values reported in our previous studies [19, 20].

The RNA transcript stability and potential alteration of the transcript level for six genes and for 13 miRNAs were investigated by qPCR. The names of the investigated six mRNAs and 13 miRNAs are presented in Additional file 1. The six genes were selected based on our previous studies [19, 20], while the 13 miRNAs were selected based on literature search [21, 22] and their expression in blood samples. The interference of the high percentage of globin transcripts from red blood cells (RBC)—constituting ~70 % of the whole blood mRNA—may decrease the sensitivity of detecting less abundant mRNA transcripts, particularly in the microarray and next-generation sequencing technologies [28, 29]. We evaluated whether low abundant RNA transcripts, i.e., transcripts with *Cq*-values above 30 cycles, could be detected in blood-RNA samples isolated using the five blood-RNA isolation protocols. Low abundant mRNA and miRNA transcripts were detected in all analyzed samples, and the results are presented in Additional file 1.

**Fig. 2:**
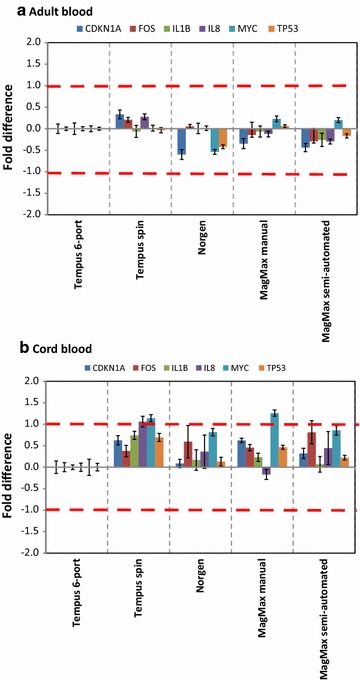
RNA transcript levels of six target genes from adult and cord blood samples. RNA isolated from adult and cord blood samples using five different protocols and analyzed by qPCR. **a** Relative transcript levels of six genes from adult blood samples collected in Tempus tubes (n = 11–12 Tempus tubes for each protocol). **b** Relative transcript levels of six genes from cord blood samples collected in Tempus tubes (n = 3–6 Tempus tubes for each protocol). The Tempus 6-port samples were used as reference samples (calibrators) and all other samples were compared against the reference samples. *Each bar* represents the average log2-transformed fold change values; fold change = 2^−∆∆*Cq*^. The *error bars* indicate ± SE and the stippled lines indicate ± twofold

For mRNA transcript stability, the non-normalized raw *Cq*-values for the six genes from adult and cord blood samples isolated with the five RNA isolation protocols are presented in Tables 3 and 4, respectively. Differences in the raw *Cq*-values between the RNA isolation protocols for each gene were small. The variations in the raw *Cq*-values were evaluated by calculating the coefficient of variation (CV) of each gene for each protocol, and the CV are presented in Tables 3 and 4, respectively. For adult blood samples, the variations in the CVs within a protocol and between protocols were small; the CVs ranged from 0.8 to 3.0 % (Table 3). For cord blood samples, the variations in the CVs within a protocol and between protocols were also small; the CVs ranged from 0.4 to 7.5 % (Table 4). The average CV value of the *IL8* gene isolated with the Norgen protocol had the highest CV value (CV = 7.5 %) (Table 4); the observed variability could not be explained from the RNA quality parameters of RNA samples isolated with Norgen protocol. Nevertheless, the calculated CV values for adult and cord blood samples were less than 10 % for all samples indicating very low variability (Tables 3, 4). The raw *Cq*-values were then normalized by the average of two stably expressed reference genes (*18S rRNA* and *GAPDH*) (Additional file 2). The normalized *Cq*-values (∆*Cq*-values) of the samples isolated with the new protocols were compared with the ∆*Cq*-values of samples isolated using the Tempus 6-port protocol (reference sample), generating fold differences (Fig. 2). This comparison was done since the Tempus 6-port protocol is a well-established RNA isolation protocol at the NIPH Biobank [19, 20]. For adult blood samples, the differences between the relative transcript levels from samples isolated using MagMax semi-automated and manual, Tempus Spin and Norgen protocols were very small when compared to samples isolated using Tempus 6-port, and the differences were less than two-fold (Fig. 2a). For cord blood samples, the relative transcript levels showed some differences (Fig. 2b); however, the transcript level changes were well within ± twofold, except for the transcript levels of *IL8* and *MYC* genes for the Tempus Spin protocol and the transcript level of *MYC* gene for the MagMax manual protocol (Fig. 2b). The reasons for the observed variable effects of RNA isolation protocols on RNA transcript stability of these genes are unclear. *IL8* and *MYC* had high average *Cq*-values above 30, particularly for the cord blood samples. The observed variability with high *Cq*-values is typical for the qPCR process and the variability increases especially in low abundant transcripts with few templates [20]. Therefore, the observed differences in the relative transcript levels of *IL8* and *MYC* genes are most likely associated with the qPCR process and may be not related to the RNA isolation protocols.

**Table 3:** Raw *Cq*-value and CVs (coefficients of variation) for adult blood samples

RNA isolation protocol	CDKN1A	FOS	IL1B	IL8	MYC	TP53
MagMax semiautomated						
Average	30.5	26.9	28.4	30.4	29.1	28.3
SD	0.7	0.2	0.8	0.3	0.4	0.4
% CV	*2.1*	*0.8*	*3.0*	*1.1*	*1.3*	*1.3*
MagMax manual						
Average	30.1	26.5	27.9	30.0	28.8	27.8
SD	0.7	0.3	0.7	0.3	0.4	0.3
% CV	*2.5*	*1.2*	*2.6*	*1.1*	*1.2*	*1.0*
Norgen						
Average	30.7	26.6	28.2	30.2	29.9	28.7
SD	0.7	0.4	0.8	0.4	0.4	0.4
% CV	*2.3*	*1.4*	*2.7*	*1.2*	*1.3*	*1.3*
Tempus spin						
Average	29.4	26.1	27.9	29.5	28.9	27.8
SD	0.7	0.3	0.8	0.4	0.3	0.4
% CV	*2.4*	*1.1*	*2.8*	*1.3*	*1.2*	*1.4*
Tempus 6-port						
Average	29.9	26.4	28.0	29.9	29.1	28.0
SD	0.8	0.2	0.7	0.3	0.3	0.3
% CV	2.5	0.8	2.4	1.0	1.1	1.0
Overall average						
Average	30.1	26.5	28.1	30.0	29.2	28.1
SD	0.9	0.4	0.8	0.5	0.5	0.5
% CV	*2.8*	*1.5*	*2.8*	*1.5*	*1.8*	*1.7*

CVs are shown in italics; the overall averaged CVs range between 1.5 and 2.8 %

**Table 4:** Raw *Cq*-value and CVs (coefficients of variation) for cord blood samples

RNA isolation protocol	CDKN1A	FOS	IL1B	IL8	MYC	TP53
MagMax semiautomated						
Average	32.5	28.0	29.8	32.2	29.9	29.5
SD	0.5	0.9	0.6	1.2	0.3	0.2
% CV	*1.4*	*3.2*	*2.0*	*3.8*	*1.1*	*0.7*
MagMax manual						
Average	*32.0*	*28.2*	29.5	32.6	29.4	29.1
SD	*0.2*	0.3	0.3	0.4	0.2	0.2
% CV	*0.6*	*1.1*	*1.1*	*1.3*	*0.7*	*0.6*
Norgen						
Average	32.7	*28.2*	29.7	31.5	30.0	29.6
SD	0.6	1.4	1.0	2.4	0.3	0.5
% CV	*1.8*	*5.1*	*3.5*	***7.5***	*1.1*	*1.6*
Tempus spin						
Average	31.8	*28.0*	28.8	31.2	29.3	28.7
SD	0.2	0.3	0.2	0.4	0.1	0.2
% CV	0.7	*1.2*	*0.7*	*1.4*	*0.4*	*0.7*
Tempus 6-port						
Average	33.0	*29.0*	30.1	32.8	31.0	29.9
SD	0.6	0.5	0.3	0.4	0.8	0.4
% CV	*1.8*	*1.8*	*0.9*	*1.3*	*2.5*	*1.2*
Overall average						
Average	32.5	28.4	29.7	32.2	30.1	29.5
SD	0.6	0.9	0.7	1.3	0.8	0.5
% CV	*2.0*	*3.0*	*2.3*	*3.9*	*2.8*	*1.8*

CVs are shown in italics; the overall averaged CVs range between 1.8 and 3.9 %. The average CV value of the IL8 gene isolated with the Norgen protocol had the highest CV value (CV = 7.5 %) and shown in bolditalics

**Fig. 3:**
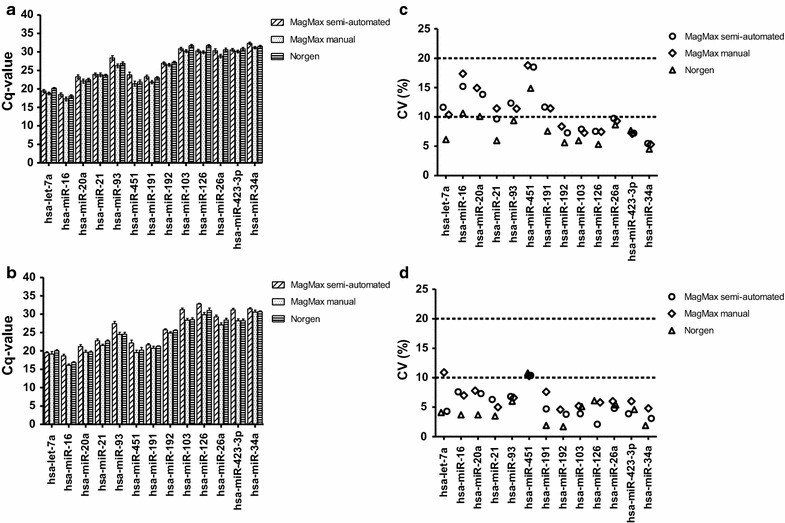
Non-normalized raw *Cq*-value and coefficients of variation of 13 miRNAs. RNA isolated from adult and cord blood using three different RNA isolation protocols optimized for simultaneous isolation of all RNA species and analyzed by qPCR. The non-normalized raw *Cq*-values for adult blood (n = 12 Tempus tubes) and cord blood (n = 3 Tempus tubes) samples collected in the Tempus tubes. **a** Average non-normalized raw *Cq*-values for adult blood samples; **b** the average non-normalized raw *Cq*-values for cord blood samples. The *error bars* indicate ± SE. There are no significant differences in the raw *Cq*-values between the three RNA isolation protocols. The coefficients of variation (CV) of the average raw *Cq*-values were calculated for adult and cord blood samples for each RNA isolation protocol for the 13 miRNAs. **c** CVs for adult blood samples (ranging from 4.5 to 18.8 %); **d** CVs for cord blood samples (ranging from 1.7 to 10.9 %). CV of 10 and 20 % is indicated by stippled lines. Each point represents the average CV of samples for one miRNA from one Tempus tubes from one protocol

Tempus 6-port and the Tempus Spin kits are not optimized for isolation of small RNAs (<200 nucleotides) and the supplier of these kits did not recommend using these two kits for small RNAs isolation. We therefore evaluated the stability of 13 miRNA transcripts from the RNA samples isolated using the three RNA isolation protocols optimized for simultaneous isolation of all RNA species; MagMAX semi-automated, MagMAX manual, and Norgen protocols. The non-normalized raw *Cq*-values and the CV values for adult and cord blood samples of the 13 miRNAs are presented in Fig. 3a–d, respectively. There were some differences in the raw *Cq*-values of the 13 miRNAs between the RNA isolation protocols (Fig. 3a, b). For both adult and cord blood samples, the CVs between protocols were not higher than the variability within a protocol (i.e., the variability between donors within a protocol); the CVs ranged from 4.5 to 18.8 % for adult blood samples and from 1.7 to 10.9 % for cord blood samples, respectively (Fig. 3c, d). For adult blood samples, the CVs for the miRNA transcripts from RNA samples isolated with Norgen protocol were in general slightly lower than with the other two protocols (Fig. 3c). An interesting general finding was that cord blood samples had a narrower CV range than adult blood samples (Fig. 3c, d), and most of the miRNA transcripts had CV values lower than 10 %, except the CV for hsa-miR451 for all three protocols and the CV for hsa-let-7a for the MagMax manual protocol (Fig. 3d). It has been reported that average CV values lower than 25 % are typically observed for stably expressed reference genes in relatively homogeneous samples [20, 30]. In this study, blood samples from several human donors were analyzed, and usually high variations are observed from heterogeneous samples such as human samples. The calculated CVs for both adult and cord blood samples were less than 20 % for all RNA isolation protocols (Fig. 3c, d), and these results suggest that the RNA isolation protocols had similar effects on the miRNA transcript stabilities (Fig. 3c, d).

**Fig. 4:**
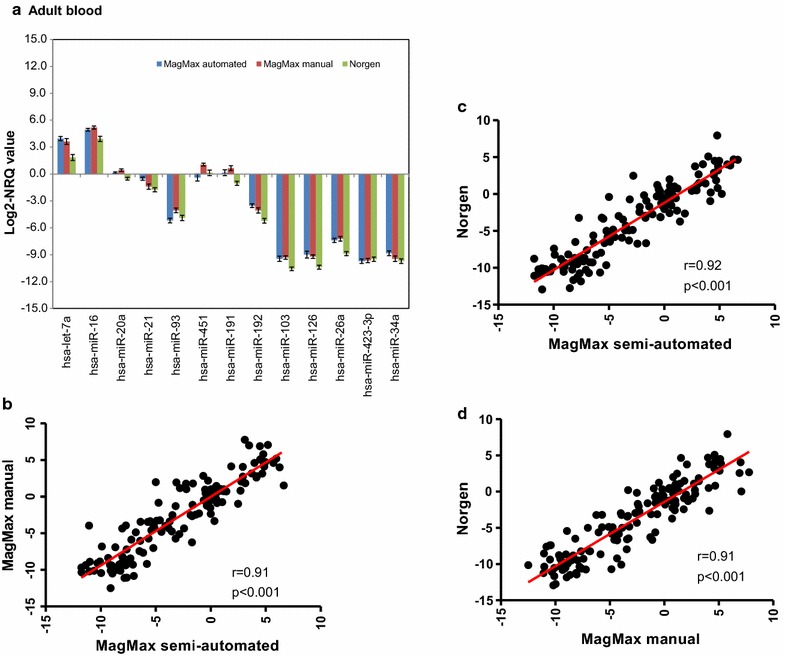
Log2-NRQ values for 13 miRNAs for adult blood samples. The normalized relative quantity values [NRQ = 2^−ΔCq^(sample); where the ΔCq (sample) = Cq (miRNA) − Cq (internal control)]) of 13 miRNAs from adult samples collected in Tempus tubes (n = 12 Tempus tubes for adult blood). **a** Log2-transformed NRQ-value of 13 miRNAs for the three RNA isolation protocols. *Each bar* represents the log2-transformed NRQ values and the *error bar* indicates ± SE; **b** correlation between MagMax semi-automated and manual protocols; **c** correlation between MagMax semi-automated and Norgen protocols; **d** correlation between MagMax manual and Norgen protocols. There are significant (p < 0.001) correlations between the NRQ-values of the three protocols. Each point represents the average of log2-NRQ values of three technical replicates from one Tempus tube, i.e., 13 miRNAs × 12 blood samples from 12 Tempus tubes

**Fig. 5:**
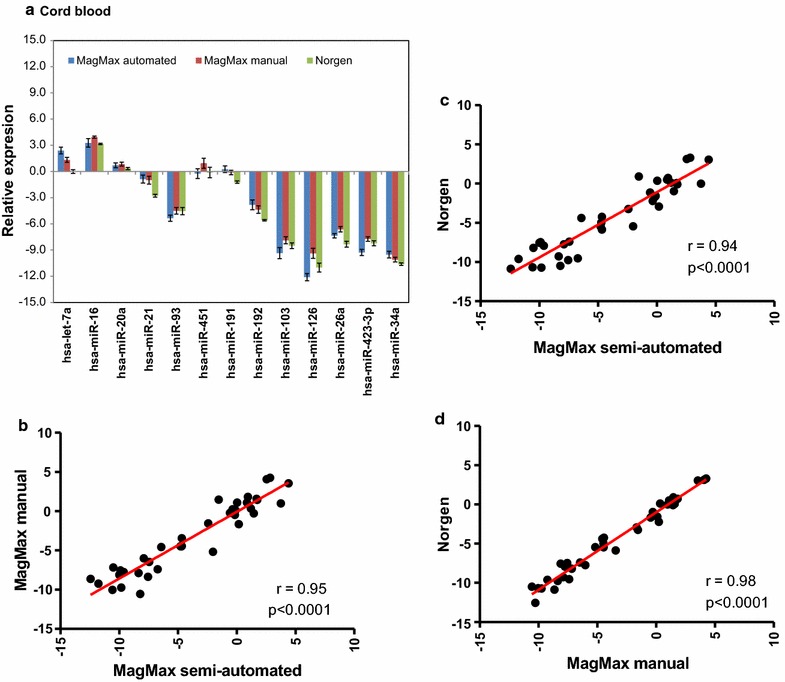
Log2-NRQ values for 13 miRNAs for cord blood samples. The normalized relative quantity values [NRQ = 2^−ΔCq^(sample); where the ΔCq (sample) = Cq (miRNA) − Cq (internal control)]) of 13 miRNAs from cord samples collected in the Tempus tubes (n = 3 Tempus tubes for adult blood). **a** Log2-transformed NRQ-value of 13 miRNAs for the three RNA isolation protocols. *Each bar* represents the log2-transformed NRQ values and the *error bar* indicates ± SE; **b** correlation between MagMax semi-automated and manual protocols; **c** correlation between MagMax semi-automated and Norgen protocols; **d** correlation between MagMax manual and Norgen protocols. There are significant (p < 0.001) correlations between the NRQ-values of the three protocols. Each point represents the average of log2-NRQ values of three technical replicates from one Tempus tube, i.e., 13 miRNAs × 3 blood samples from 3 Tempus tubes

The raw *Cq*-values of the 13 miRNAs were then normalized by the average of three stably expressed reference small nuclear RNAs (*RNU6*, *RNU43* and *RNU1*) (Additional file 3), and the average log2 normalized relative quantity (log2- NRQ) values are presented in Figs. 4a, 5a. For adult blood samples, some differences in the average transcript levels of some miRNAs were observed between the three RNA isolation protocols (Fig. 4a). The most pronounced difference was observed between Norgen protocol on one hand and the other two MagMax protocols on the other hand (Fig. 4a). This was not an unexpected finding, since MagMax semi-automated and manual protocols share similar kit components. Nevertheless, high and significant correlations between the log2-NRQ values of the 13 miRNAs between the MagMax semi-automated and the MagMax manual protocols (r = 91.3 %; p < 0.001), between the MagMax semi-automated and the Norgen protocols (r = 92.2 %; p < 0.001), and between the MagMax manual and the Norgen protocols (r = 91.1 %; p < 0.001), were observed (Fig. 4b–d).

Similar conclusions can be drawn for cord blood samples (Fig. 5a), where the average log2-NRQ-values of some miRNAs were differentially expressed between the three RNA isolation protocols (Fig. 5a). As with adult blood samples, high and significant correlations were observed between the three RNA isolation protocols (Fig. 5b–d). The correlation between the MagMax semi-automated and the MagMax manual protocols was r = 95.0 %; p < 0.0001, whereas the correlation between the Norgen protocol and the MagMax semi-automated or the MagMax manual protocols were r = 94.0 %; p < 0.0001 and r = 98.0 %; p < 0.0001, respectively (Fig. 5b–d). Taken together, the small variability in the raw *Cq*-values and the high correlation of NRQ-values demonstrate that the three RNA isolation protocols could be used interchangeably.

Among the two well-established commercially available blood-RNA stabilizing platforms (Tempus Blood RNA and PAXgene™ Blood RNA system) where blood is drawn directly into a tube containing RNA stabilizing reagents, the PAXgene™ Blood RNA system is an established system for the isolation and analysis of small RNAs (particularly, miRNAs) [31, 32]. A literature search in the publicly available databases revealed no per-reviewed reports on small ncRNAs quality and transcript stability from cord blood samples collected in the Tempus tubes. This report is therefore the first systematic evaluation of the stability of small ncRNA (particularly, miRNAs) in cord samples collected in Tempus tubes.

Other aspects to consider when choosing a new method are cost (cost per sample) and sample processing time. We calculated the cost per sample for each protocol and the RNA processing time (i.e., RNA isolation time plus hands-on-time, including centrifugation, vortexing, optional DNase treatment step and incubating time) for six Tempus tubes (Table 5). Six Tempus tubes were analyzed because only six samples can be processed on the Tempus 6-port protocol at the same time. There were differences in the cost per sample between the protocols; Norgen is the cheapest kit whereas Tempus 6-port is the most expensive kit (Table 5). The RNA processing cost is particularly important for large-scale human biobanks. At NIPH Biobank, more than 50,000 adult and cord blood samples were collected in the Tempus tubes and stored. It is likely that RNA will be isolated from all of these blood samples. If all the 50,000 blood samples are processed, a difference by only one US dollar in a sample processing cost between two protocols will be large. It is therefore very important to carefully consider this aspect before a final choice of RNA isolation protocol. We noted some differences in the RNA processing time between the protocols. Semi-automation of the RNA isolation process did not significantly reduce the overall sample processing time (Table 5).

**Table 5:** RNA isolation protocols costs and sample processing times

RNA isolation protocol	Cost per sample^a^	RNA processing time for six samples (min)^c^
MagMAX semi-automated	18.5^b^	90
MagMAX manual	12.7^b^	120
Norgen	10.5	90
Tempus Spin	14.0	90
Tempus 6-port	29.0^b^	120

^a^The cost of kits and other consumables, in US Dollars

^b^Price does not include the cost of MagMAX™ Express 96 deep-well Magnetic Particle Processor for MagMax semi-automated, the cost of 96 well Magnetic-Ring Stand for MagMax manual, and the cost of ABI PRISM™ 6100 Nucleic Acid PrepStation for Tempus 6-port

^c^RNA processing time for six samples in the authors’ laboratory—it includes RNA isolation time and hands-on-time such as centrifugation, vortexing, incubation etc

**Fig. 6:**
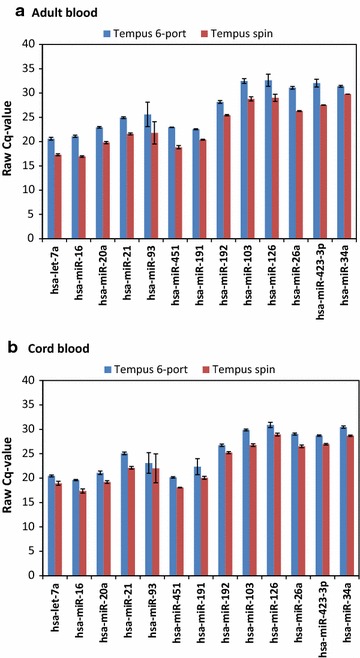
Non-normalized raw *Cq*-value for Tempus spin and 6-port. The non-normalized raw *Cq*-values for adult blood and cord blood samples collected in the Tempus tubes. **a** Average non-normalized raw *Cq*-values for adult blood samples (three replicates from pooled RNA samples per protocol); **b** average non-normalized raw *Cq*-values for cord blood samples (three replicates from pooled RNA samples per protocol). The *error bars* indicate ± SE

Finally, we evaluated the possibility of detecting miRNA transcripts from RNA samples isolated using the Tempus spin and the Tempus 6-port protocols. The supplier of the two kits did not recommend the use of these kits for isolation of small RNAs (<200 nucleotides). We therefore analyzed pooled RNA samples resulting from the two protocols. Each pooled RNA sample was divided into three equal aliquots for each adult and cord blood samples; the transcript levels of the 13 miRNAs were then analyzed by qPCR (Fig. 6). The average raw *Cq*-values for the 13 miRNAs obtained both from adult and cord blood samples from the Tempus spin and the Tempus 6-port RNA isolation protocols were within the range of the average *Cq*-values obtained from RNA samples from the other three protocols (MagMax semi-automated, MagMax manual, and Norgen protocols) optimized for small RNA isolation (Fig. 6a, b). Even though these two protocols were optimized for isolation of large RNA molecules (i.e., >200 nucleotides) and not for RNA of a smaller size range, it seems that comparable miRNA transcript levels can be detected from these protocols (Fig. 6a, b). The presence of one peak for each miRNA amplicon during melting curve analysis indicates that a single amplicon has been generated by the qPCR assay. We observed one single peak for each miRNA from the RNA samples isolated with the Tempus spin and the Tempus 6-port protocols (data not shown). The observed amplicon peak for each miRNA was similar to the peak observed from the RNA samples isolated with the other three protocols indicating the absence of non-specific amplification. Successful detection of miRNA transcripts from RNA samples isolated with the Tempus spin and the Tempus 6-port protocols was unexpected and represents an interesting finding; however, these two kits are not among the cheapest blood-RNA isolation protocols optimized for blood samples collected in the Tempus tubes (Table 5).

## Conclusions

Our results indicate that high-quality total RNA, including miRNAs, suitable for mRNA and miRNA expression profile analysis, can be obtained from blood samples collected into Tempus tubes using different commercially available RNA isolation protocols. A balanced consideration of the RNA yield, quality, transcript stability, sample processing time and cost will have a large influence on the choice of RNA isolation protocol. The integrity and purity of the blood-RNA isolated from these RNA isolation protocols were comparable and they were of high quality. In general, the mRNA transcript levels of the six studied genes were relatively stable. The different RNA isolation protocols did not show a significant influence on their transcript stability. However, the miRNA transcript levels were slightly affected by the different RNA processing methods, although the 13 miRNAs had similar expression direction and their levels were within the same order of magnitude. The most unexpected and interesting finding was detection of miRNA transcripts from RNA samples isolated with the Tempus spin and the Tempus 6-port protocols, originally not optimized for isolation of small RNAs. Overall, a satisfactory amount of high-quality RNA was obtained from all five protocols suitable for mRNA and miRNA expression profiling; the Tempus blood RNA system seems to be robust and flexible and not to be dependent on one specific RNA isolation method. This is good news for large-scale human biobanks such as the NIPH Biobank, where tens of thousands of samples are collected in Tempus tubes and stored for future use.

### Methods

#### Sample collection and experimental design

Whole blood samples were collected from four healthy, consenting adult volunteers among the NIPH staff. Umbilical cord blood samples were collected from six newborns whose mothers had given their informed consent to participate in MoBa. The samples were collected into Tempus tubes (3 ml blood per tube) according to the manufacturer’s instructions (Life Technologies, Norway). The Tempus tubes were from the same lot number. In total, 59 tubes from four adults and 18 tubes from six newborns were collected. The tubes were randomized for each donor and labelled with unique numbers. Each Tempus tube was considered as an independent biological sample. The adult blood samples were kept at room temperature for 2 h before freezing at −20 °C overnight and then transferred to −80 °C until processing. The cord blood samples were collected at the maternity unit at Ullevål University Hospital and shipped to NIPH at ambient temperature within 1 day, and then handled in the same way as samples from the adults. The cord blood samples are part of blood samples collected specifically for RNA QC for cord blood samples stored at MoBa. For each protocol, 12 adult blood samples and 3 cord blood samples were analyzed, except for the Tempus 6-port protocol, where 11 adult blood and 6 cord blood samples were analyzed. With each Tempus tube considered as an independent biological sample, the analysis of in total 77 Tempus tubes should provide a good estimate of the quality and stability of the samples. MoBa has obtained its license from the Norwegian Data Inspectorate (01/4325) and the MoBa project is approved by the Regional Committee for Medical Research Ethics (S-97045, S-95113); participants gave their informed consent.

#### RNA extraction protocols

Total RNA from blood collected in Tempus tubes was extracted using four different commercial kits: MagMAX for Stabilized Blood Tubes RNA Isolation Kit (compatible with Tempus Blood RNA Tubes) (Life Technologies, Norway); Tempus Spin RNA Isolation kit; Tempus 6-port RNA isolation kit on an ABI PRISM TM 6100 Nucleic Acid Prep Station (Life Technologies, Norway); and finally, Preserved Blood RNA purification kit 1 (for use with Tempus Blood RNA Tubes) (Norgen Biotek Corp, Canada). For the MagMAX kit, RNA was isolated according to the manufacturer’s protocol which included a TURBO DNase and protease step. Both a manual and a semi-automated protocol were used for the isolation of RNA. For the manual protocol, samples were processed using 1.5-ml microfuge tubes on a magnetic stand; for the semi-automated protocol, a 96-well processing plate on a MagMAX Express-96 Magnetic Particle Processor was used. In the washing steps, twice the amount of washing solution, e.g. 300 µl, was used. Tempus tubes were processed using Tempus 6-port RNA isolation kit as previously described. For the Norgen Preserved Blood RNA Purification kit I, the Tempus 6-port RNA isolation kit, and the Tempus Spin RNA isolation kit, optional DNase treatments were included in accordance with each manufacturer’s protocol. The isolated total RNA was stored at −80 °C until analysis in elution buffers supplied with each respective RNA isolation kit.

#### RNA QC

The concentration of extracted total RNA was measured using NanoDrop ND-8000 spectrophotometer (Thermo Scientific, Norway). RNA purity was estimated by examining the OD 260/280 and the OD 260/230 ratios. RNA integrity was found using the Eukaryote total RNA 6000 Nano LabChip kit and Eukaryote total RNA Nano assay on an Agilent 2100 Bioanalyzer according to the manufacturer’s instructions (Agilent Technologies, Norway). RNA integrity numbers (RIN) from 1 to 10 (low to high RNA quality) were calculated using the 2100 Expert software (Agilent Technologies, Norway).

#### Quantitative real time PCR (qPCR) assay

The cDNA synthesis was performed with 100 ng total RNA from samples as template, using the High Capacity cDNA Reverse Transcription Kit (Life Technologies, Norway) according to the manufacturer’s protocol. The amplification reactions were carried out in a DNA Engine DYAD Peltier Thermal Cycler (BioRad, Norway) with the following steps: 10 min at 25 °C, 2 h at 37 °C and finally, 5 min at 85 °C. For the microRNA (miRNA) study, cDNA from 1 µg of RNA was synthesized using the miScript II RT kit including 5× miScript HiSpec Buffer (for selective conversion of mature miRNA into cDNA) according to the manufacturers protocol (Qiagen, Norway). A no reverse transcriptase control (NRT) was included and samples were incubated at 37 °C for 60 min and 95 °C for 5 min. All cDNA samples were stored at −20 °C prior to gene expression analysis.

Quantitative real-time PCR (qPCR) was carried out in 96-well PCR plates using TaqMan Fast Universal PCR Master Mix, No AMpErase UNG, according to the manufacturer’s protocol (Life technologies, Norway) on a Fast 7500 Real Time PCR system (Life Technologies, Norway). The following cycling conditions were used: an enzyme activation step at 95 °C for 20 s, and then 40 cycles of annealing and extension steps at 95 °C for 3 s and 60 °C for 30 s, respectively. Serial dilutions of cDNA were prepared to determine the appropriate cDNA dilution. A 1:10 dilution of cDNA from each Tempus tube was run in triplicate for each gene of interest. Non-template controls (NTC) were included in all assays. Transcript levels for the following six genes were measured as described in our recent study [20], applying commercial primers and probe assays from Life Technologies: *CDKN1A* (PN: Hs00355782_m1), *FOS* (PN: Hs00170630_m1), *IL1B* (PN: Hs00174097_m1), *IL8* (PN: Hs001700174103_m1), *MYC* (PN: Hs00153408_m1) and *TP53* (PN: Hs00153340_m1), *18S rRNA* (PN: Hs99999901) and *GAPDH* (PN: Hs9999905_m1). The geometric average of the two reference genes*, 18S rRNA* and *GAPDH* was used for normalization. The six genes were selected based on our recent study [19] and literature search [33, 34], with mRNA transcript abundance from low to high abundant targets (Additional file 1).

MicroRNA specific qPCR was carried out in 384-well PCR plates using miScript SYBR Green PCR Kit according to the manufacturer’s protocol (Qiagen, Norway) on a CFX384 Touch™ Real-Time PCR Detection System (Bio-Rad, Norway). Serial dilutions of cDNA were prepared to determine the optimal dilution. A 1:10 dilution of cDNA from each Tempus tube was run in triplicate for each gene of interest. The cycling program included an initial enzyme activation step at 95 °C for 15 min, and then 40 cycles of denaturation, annealing and extension steps at 94 °C for 15 s, 55 °C for 30 s and 70 °C for 30 s, respectively. The melting curve (T_m_) analysis was included in each run. Non-template controls (NTC) and non-reverse transcriptase controls (NRT) were included in each run. The expression levels of the following 13 miRNAs (*hsa*-*let*-*7a, hsa*-*miR*-*16, hsa*-*miR*-*20a, hsa*-*miR*-*21, hsa*-*miR*-*26a, hsa*-*miR*-*34a, hsa*-*miR*-*451, hsa*-*miR*-*93, hsa*-*miR*-*103, hsa*-*miR*-*126, hsa*-*miR*-*191, hsa*-*miR*-*192, hsa*-*miR*-*423*-*3p*), and three small nuclear RNAs (snRNA; *RNU6*, *RNU43* and *RNU1*), were measured. These miRNAs were selected based on their expression abundance in blood and literature search [21, 22].

#### Data analysis

The quantification cycle (*Cq*) values were recorded with SDS v1.3 software (Life Technologies, Norway) or CFX Manager™ Software (Bio-Rad, Norway). The raw *Cq*-values were then exported into Excel-files and analyzed by the comparative *Cq*-method [35, 36] using *18S rRNA* and *GAPDH* as reference genes (internal control) for mRNA transcript level analysis, while *RNU6*, *RNU43* and *RNU1* were used as reference genes for miRNA transcript level analysis. Prior to normalization, the raw data (*Cq*-values) generated from qPCR experiments were pre-processed to ensure that measurements at low levels were well within the linear area of detection; *Cq*-values radically different from other technical replicates were classified as outliers and excluded. In addition, all *Cq*-values above 35 were considered beyond the limit of detection (LOD) and coded as missing values, because *Cq*-values above 35 cycles are in general not reliable. Target genes were normalized by the average of stably expressed reference genes, [this is given by Δ*Cq*; where ∆*Cq* (sample) = *Cq* (target gene) − *Cq* (reference genes)]. The ΔΔ*Cq* values were generated by subtracting the Δ*Cq*-value for the reference samples (calibrators; Tempus 6-port) from the Δ*Cq*-value for the samples [∆∆*Cq* = ∆*Cq* (sample) − ∆*Cq* (calibrator); fold change = 2^−∆∆*Cq*^]. For miRNA data, the target miRNAs were normalized by the average of three stably expressed reference genes (*RNU6*, *RNU43* and *RNU1*); this is given by Δ*Cq*; where ∆*Cq* (sample) = *Cq* (target miRNA) − *Cq* (reference snRNAs). The ∆*Cq* values were then presented as normalized expression (NRQ = 2^−∆*Cq*^). The NRQ values were then log2-transformed in order to make the values symmetrical around zero. The reference gene stability was evaluated and results are presented in Additional files 2 and 3.

#### Statistical analysis

Statistical analysis of RNA yield, purity, integrity and ∆*Cq*-values was carried out by one-way analysis of variance (ANOVA), followed by post hoc Tukye’s HSD (honest significant difference) tests to allow for multiple comparisons or by non-parametric Kruskal–Wallis test. Normal distribution and equality of variances were evaluated using the Shapiro–Wilk normality test and the Levene’s or Bartlett test of homogeneity of variance. The correlations of the log2-NRQ values of the 13 miRNAs between the three RNA isolation protocols (MagMax semi-automated, MagMax manual and Norgen) were analyzed by Pearson or Spearman statistics and the regression lines were generated using GraphPad Prism 5.0 (GraphPad Software, Inc., USA). Statistical analyses were performed using the R statistical programming environment (version 3.1.1), and results with p < 0.05 were accepted as statistically significant. The data are presented as mean ± SE. The coefficient of variation (CV) of the non-normalized raw *Cq*-value for each gene was calculated by dividing the mean *Cq*-value with the standard deviation. CV, expressed as a percentage, was used for computing the degree of variation in the average *Cq*-values of the RNA isolation protocols.

## Additional files

10.1186/s13104-016-2224-y Target abundance of RNA transcripts. Evaluation of target abundance; i.e., whether low abundant RNA transcripts (RNA transcripts with Cq-values above 30 cycles) could be detected in blood-RNA samples isolated using the five blood-RNA isolation protocols. Low abundant mRNA and miRNA transcripts were detected in all analyzed samples. The average of non-normalized raw *Cq*-values of all targets from all adult (n=11-12 Tempus tubes) and cord blood (n=3-6 Tempus tubes) samples collected in the Tempus tubes of the blood-RNA samples isolated using the five RNA isolation protocols.
10.1186/s13104-016-2224-y Evaluation of reference gene stability. The non-normalized raw *Cq*-values of reference genes for adult (n=11-12 Tempus tubes) and cord blood (n=3-6 Tempus tubes) samples collected in the Tempus tubes of the blood-RNA samples isolated using the five RNA isolation protocols.
10.1186/s13104-016-2224-y Evaluation of reference gene stability. The non-normalized raw *Cq*-values of reference small ncRNAs for adult (n=12 Tempus tubes) and cord blood (n=3 Tempus tubes) samples collected in the Tempus tubes of the blood-RNA samples isolated using the three RNA isolation protocols.

## Abbreviations

*Cq*: quantification cycle threshold
MoBa: the Norwegian Mother and Child Cohort Study
NIPH: Norwegian Institute of Public Health
ncRNA: non-coding RNA
miRNA: microRNA
OD: optical density
qPCR: quantitative real-time PCR
RIN: RNA integrity number
