# Supplementary material for: Comparison of blood RNA isolation methods from samples stabilized in Tempus tubes and stored at a large human biobank
Source: BMC Res Notes. 2016 Sep 1;9(1):430. doi: 10.1186/s13104-016-2224-y (PMC5009671; doi:10.1186/s13104-016-2224-y)
Supplement: Supplementary file 1 — 10.1186/s13104-016-2224-y Target abundance of RNA transcripts. Evaluation of target abundance; i.e., whether low abundant RNA transcripts (RNA transcripts with Cq-values above 30 cycles) could be detected in blood-RNA samples isolated using the five blood-RNA isolation protocols. Low abundant mRNA and miRNA transcripts were detected in all analyzed samples. The average of non-normalized raw Cq-values of all targets from all adult (n=11-12 Tempus tubes) and cord blood (n=3-6 Tempus tubes) samples collected in the Tempus tubes of the blood-RNA samples isolated using the five RNA isolation protocols. [file 13104_2016_2224_MOESM1_ESM.pdf]

Additional file 1

Title: Target abundance of RNA transcripts

Evaluation of target abundance; i.e., whether low abundant RNA transcripts (RNA transcripts with Cq-values above 30 cycles) could be detected in blood-RNA samples isolated using the five blood-RNA isolation protocols. Low abundant mRNA and miRNA transcripts were detected in all analyzed samples. The average of non-normalized raw *Cq*-values of all targets from all adult (n=11-12 Tempus tubes) and cord blood (n=3-6 Tempus tubes) samples collected in the Tempus tubes of the blood-RNA samples isolated using the five RNA isolation protocols.

Table 1. Target abundance of RNA transcripts

Gene name	Average Cq ± SD
**High abundant target (Cq < 25)**	
hsa-let-7a	18.31 ± 1.95
hsa-miR-16	16.87 ± 2.65
hsa-miR-20a	23.44 ± 2.99
hsa-miR-21	24.86 ± 2.20
hsa-miR-191	21.61 ± 2.50
hsa-miR-451	23.17 ± 4.04
**Medium abundant target (25 ≤ Cq ≤ 30)**	
FOS	28.39 ± 0.85
IL1B	29.65 ± 0.68
TP53	29.46 ± 0.54
hsa-miR-26a	29.26 ± 3.44
hsa-miR-93	28.27 ± 3.21
hsa-miR-192	25.54 ± 1.93
**Low abundant target (Cq > 30)**	
CDKN1A	32.48 ± 0.64
IL8	32.19 ± 1.26
MYC	30.08 ± 0.83
hsa-miR-34a	31.24 ± 2.10
hsa-miR-103	31.46 ± 3.21
hsa-miR-126	30.27 ± 2.63
hsa-miR-423-3p	30.30 ± 3.08
