# Supplementary material for: Comparison of blood RNA isolation methods from samples stabilized in Tempus tubes and stored at a large human biobank
Source: BMC Res Notes. 2016 Sep 1;9(1):430. doi: 10.1186/s13104-016-2224-y (PMC5009671; doi:10.1186/s13104-016-2224-y)

Additional file 3

File format: PDF

Title: Evaluation of reference gene stability

The non-normalized raw *Cq*-values of reference small ncRNAs for adult (n=12 Tempus tubes) and cord blood (n=3 Tempus tubes) samples collected in the Tempus tubes of the blood-RNA samples isolated using the three RNA isolation protocols.

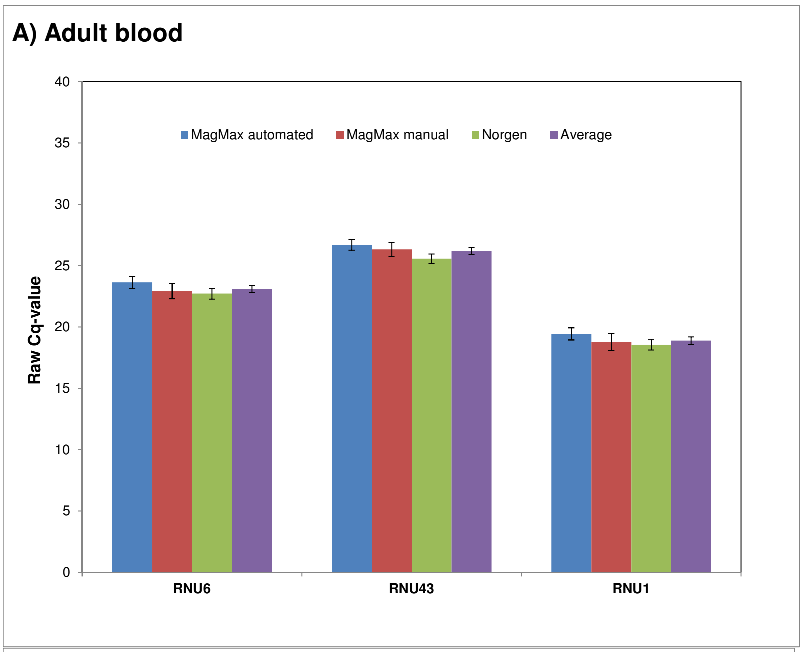

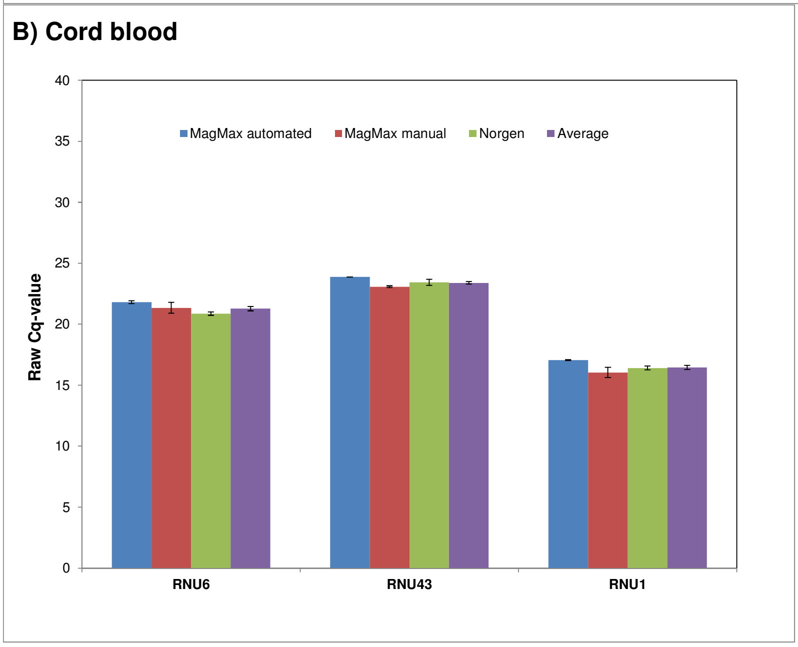

Supplement: Supplementary file 3 — 10.1186/s13104-016-2224-y Evaluation of reference gene stability. The non-normalized raw Cq-values of reference small ncRNAs for adult (n=12 Tempus tubes) and cord blood (n=3 Tempus tubes) samples collected in the Tempus tubes of the blood-RNA samples isolated using the three RNA isolation protocols. [file 13104_2016_2224_MOESM3_ESM.pdf]
